# Comorbidity-Adjusted Survival in Early Stage Lung Cancer Patients Treated with Hypofractionated Proton Therapy

**DOI:** 10.1155/2010/251208

**Published:** 2010-12-01

**Authors:** Sharon Y. Do, David A. Bush, Jerry D. Slater

**Affiliations:** Department of Radiation Medicine, Loma Linda University Medical Center, 11234 Anderson Street, Loma Linda, CA 92354, USA

## Abstract

*Objective*. To determine the influence of comorbidity on survival in early-stage lung cancer patients treated with proton radiotherapy, using the Charlson Comorbidity Index. *Study Design and Setting*. Fifty-four non-small-cell lung cancer patients, treated prospectively in a phase II clinical trial with hypofractionated proton therapy, were analyzed retrospectively to assess their burden of comorbid disease as expressed by Charlson Comorbidity Index. Using the Charlson Comorbidity Index method, a predicted survival curve based on comorbidity was formulated and compared to the observed mortality from causes other than lung cancer in the study population. *Results*. The study population had an average age score of 2.8 and an average Charlson Comorbidity Index of 4.7. Predicted survival was calculated to be 67% and 50% at 2 and 4 years, respectively. Actual comorbidity-specific survival at 2 and 4 years was 64% and 45%, respectively. The observed survival fell within the 95% confidence intervals of the predicted survival at all time points up to 5 years. *Conclusion*. Predicted mortality from concurrent disease, based on Charlson Comorbidity Index, correlated well with observed comorbidity-specific mortality. This helps substantiate the accuracy of the data coding in cause of death and strengthens previously reported disease-specific survival rates.

## 1. Introduction

More people die from lung cancer than from any other type of cancer. It continues to be the leading cause of cancer death in both men and women, with an estimated 161, 840 deaths in 2008 [1]. The patient population affected by lung cancer is generally older: the average age is 71 years at the time of diagnosis [1]. A higher prevalence of comorbidity is associated with age; thus, lung cancer patients tend to have more comorbidities. Additionally, given the causative association of tobacco smoking, chronic pulmonary and cardiovascular diseases are commonly seen in this patient population. 

In oncological studies, efficacy of treatment is often evaluated in terms of survival outcome. This can be problematic in lung cancer, as the high incidence of concomitant illnesses exerts a confounding influence on assessing survival outcome. Many studies exploring nonsurgical treatment in early stage lung cancer report promising disease-specific survival (DSS), but overall survival (OS) remains poor. Fakiris et al. reported a 3-year DSS of 81.7% in early stage non-small-cell lung cancer (NSCLC) patients treated with stereotactic body radiation (SBRT), but an OS of 42.7% [2]. Salazar explored once-weekly, high-dose SBRT in inoperable patients, which included 60 stage I patients; the 5-year DSS and OS rates were 70% and 47%, respectively [3]. A study conducted in Japan by Uematsu et al., also exploring SBRT, reported 3-year DSS and OS rates of 88% and 66%, respectively, [4]. The disparity between DSS and OS can be explained by advanced age and high mortality from comorbid illnesses in this patient population. 

It is clear that disease-specific survival is a more appropriate endpoint than overall survival in examining therapeutic outcome in lung cancer patients. However, determining DSS is not entirely exempt from subjectivity. The pathophysiology of multiple comorbidities in lung cancer patients are integrated, and death attributable to a single specific cause can be difficult to ascertain. Determining the cause of death requires a judgment call that cannot always be made objectively. This paper employs the Charlson Comorbidity Index (CCI) to elucidate the influence of comorbidity. Using the Charlson model, a predicted survival curve can be formulated based on patients' baseline medical problems [5]. In other words, the life expectancy of a specific cohort of patients, based on their comorbidities, can be estimated. This study uses the CCI tool and predicted survival as an alternative statistical analysis to validate previously published results of hypofractionated proton beam radiotherapy for stage I NSCLC [6].

## 2. Methods

**Figure 1:**
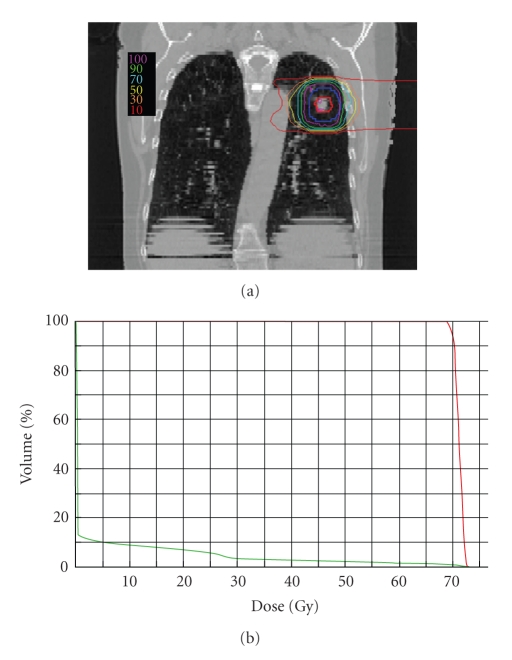
(a) Representative treatment plan. Colored contour lines indicate percentage of total dose given. (b) Dose-volume histogram showing doses given to volumes of tumor (red) and lung (green).

Patients evaluated for this report were treated at Loma Linda University Medical Center under an IRB-approved phase II protocol utilizing proton beam radiotherapy [6]. Informed consent was obtained from all subjects, who had a histologic diagnosis of NSCLC and clinical stage I disease; all were medically inoperable or refused surgical resection. Each patient received a two-week course of proton radiation, delivered in 10 fractions, of either 51 or 60 CGE to the target volume. The proton dose was calculated to a point in the center of the tumor and the 95% isodose line completely covered the target volume. The mediastinum was not treated. A representative treatment plan, including isodose distribution, is shown in Figure 1. 

Charts for 54 of these patients were reviewed, and each patient was assigned a CCI score based on age and pre-existing illnesses. Comorbibities for each patient were obtained from the initial consultation and clinical data obtained throughout followup. The average score was determined for the entire cohort, and a representative predictive survival curve for the cohort was generated using the same model as in the validation phase of the original Charlson paper [5]. Relative risk was calculated from the proportional hazards model used to create a single prognostic variable combining age and comorbidity; a value indicative of subsequent risk was obtained using the equation

(1) $$e^{0.9(\text{comorbidity + age score})}\;=\;\text{RR}.$$

Predicted survival was calculated based on a theoretical low-risk population, using the equation

(2) $$S_{o}^{\text{RR}}\;\;=\;\text{predicted survival},$$

where *S*_*o*_ is the survival of the theoretical low-risk population at a given time point and RR is the relative risk. 

The predicted survival curve based on the patient's Charlson score was then compared to the observed comorbidity-specific survival (CSS). Since the objective is to compare the observed mortality due to comorbidity with the CCI-based predicted mortality, patients who died from their lung cancers were treated as withdrawn alive, or censored, at the time of death. CSS was calculated using the Kaplan-Meier method, which is reflective of the difference between overall survival and disease-specific survival.

## 3. Results

Chronic pulmonary disease was the most prevalent comorbidity, affecting 74% of the patients evaluated; 50% of the patients had multiple comorbid illnesses. Only the illnesses that were found to be prognostic for mortality and part of the Charlson weighted index were included. The weighted index of comorbidity and the number of patients in our cohort with the condition are listed in Table 1. The CCI score incorporates the patient age score, which is computed by assigning a point for each decade over age 40. The average age of the patients was 72 years, the average age score was 2.8 years, the average CI score was 1.9, and the average CCI was 4.7.

**Figure 2:**
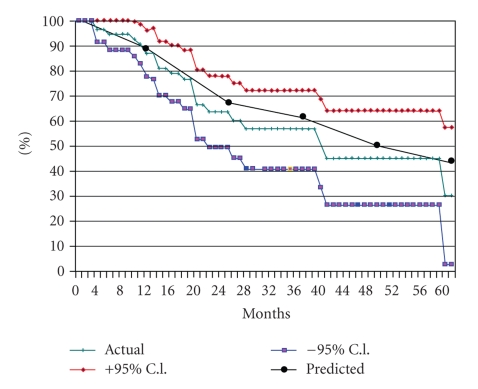
Survival curves showing actual comorbidity-specific survival and predicted survival, with 95% confidence intervals.

Predicted survival and observed comorbidity-specific survival correlated well. At 3 years the predicted survival we would expect based on the Charlson comorbidity index is 62%, and the observed 3-year CSS is 57% (Figure 2). At all time points, the two survival curves correlated well. The predicted curve lies within the 95% confidence interval, indicating no statistically significant difference between observed CSS and the predicted curve.

## 4. Discussion

The Charlson index is a weighted value that takes into account the number and severity of comorbid diseases and allows calculation of a risk score for individual patients that is prognostic for mortality. The CCI has been validated in numerous oncology studies and is widely used as a prognostic tool. Birim et al. examined NSCLC patients treated with resection to validate the influence of CCI on outcome and found that CCI is strongly correlated with higher risk of surgery and is a better predictor than individual risk factors [7]. Hall et al., in investigating the impact of age and comorbidity on survival outcomes and treatment patterns in prostate cancer, advocated the use of CCI in research outcomes and treatment decision-making [8]. Finding that CCI has been widely used and validated throughout the oncology literature, the same authors developed an electronic application for rapidly calculating CCI score, making the tool easier to use in clinical research [9]. Using the Charlson model, a predicted survival curve can be formulated to estimate the expected baseline mortality due to a patient's comorbidity. This method provides an objective means to assess life expectancy based on patients' concurrent medical illness aside from the index disease of interest, in this case, lung cancer. 

Due to the high prevalence of comorbidity associated with age and tobacco use in lung cancer patients, optimizing definitive radiotherapy as alternative treatment for medically inoperable patients with early stage disease is an important area of investigation. Historically the experience with conventional radiotherapy for early stage NSCLC has been disappointing, with a poor 3-year overall survival rate of approximately 32% and disease-specific survival of approximately 43% [10, 11]. However, in patients treated with hypofractionated proton beam therapy, the results are more favorable. A disease-specific survival rate of 72% at 3 years has been recorded. The dose distribution properties of proton therapy confer considerable advantages in focal targeting of tumor while minimizing dose to the surrounding normal tissues; studies have demonstrated reduced doses to normal tissues in lung cancer patients when compared to X-ray-based treatments [12]. The improved outcomes are likely attributable to superior tissue targeting and the enhanced biological effect of hypofractionation. Inasmuch as the results of the present study show that the predicted mortality from concurrent disease based on the CCI correlated well with the observed comorbidity-specific mortality, this helps substantiate the accuracy of the data coding in cause of death and, in turn, strengthens the validity of the reported disease-specific survival rate obtained with hypofractionated proton radiation.

## Figures and Tables

**Table 1:** Weighted index of comorbidity.

Assigned weights for diseases	Conditions	No. of patients with condition
1	Myocardial infarct	6
	Congestive heart failure	8
	Peripheral vascular disease	6
	Cerebrovascular disease	7
	Dementia	0
	Chronic pulmonary disease	40
	Connective tissue disease	3
	Ulcer disease	2
	Mild liver disease	0
	Diabetes	9
2	Hemiplegia	0
	Moderate or severe renal disease	1
	Diabetes with end organ damage	0
	Any tumor	8
	Leukemia	1
	Lymphoma	0
3	Moderate or severe liver disease	0
6	Metastatic solid tumor	0
	AIDS	0
